# Modelling a Historic Oil-Tank Fire Allows an Estimation of the Sensitivity of the Infrared Receptors in Pyrophilous *Melanophila* Beetles

**DOI:** 10.1371/journal.pone.0037627

**Published:** 2012-05-21

**Authors:** Helmut Schmitz, Herbert Bousack

**Affiliations:** 1 Institut für Zoologie der Universität Bonn, Bonn, Germany; 2 Peter Grünberg Institut, Forschungszentrum Jülich GmbH, Jülich, Germany; The Australian National University, Australia

## Abstract

Pyrophilous jewel beetles of the genus *Melanophila* approach forest fires and there is considerable evidence that these beetles can detect fires from great distances of more than 60 km. Because *Melanophila* beetles are equipped with infrared receptors and are also attracted by hot surfaces it can be concluded that these infrared receptors are used for fire detection.

The sensitivity of the IR receptors is still unknown. The lowest threshold published so far is 0.6 W/m^2^ which, however, cannot explain the detection of forest fires by IR radiation from distances larger than approximately 10 km. To investigate the possible sensitivity of the IR receptors we assumed that beetles use IR radiation for remote fire detection and we made use of a historic report about a big oil-tank fire in Coalinga, California, in 1924. IR emission of an oil-tank fire can be calculated by “pool fire” simulations which now are used for fire safety and risk analysis. Assuming that beetles were lured to the fire from the nearest forests 25 and 130 km away, our results show that detection from a distance of 25 km requires a threshold of the IR receptors of at least 3×10^−2^ W/m^2^. According to our investigations most beetles became aware of the fire from a distance of 130 km. In this case the threshold has to be 1.3×10^−4^ W/m^2^. Because such low IR intensities are buried in thermal noise we suggest that the infrared sensory system of *Melanophila* beetles utilizes stochastic resonance for the detection of weak IR radiation. Our simulations also suggest that the biological IR receptors might be even more sensitive than uncooled technical IR sensors. Thus a closer look into the mode of operation of the *Melanophila* IR receptors seems promising for the development of novel IR sensors.

## Introduction

Jewel beetles of the genus *Melanophila* make use of a very special ecological niche for reproduction: freshly burnt trees [1], [2], [3]. Immediately after a fire has raged over a forest, beetles of both sexes can be found on the burnt area starting reproduction [4], [5], [6], [7]. While doing so these highly pyrophilous insects are well protected by the smoke and heat given off by remnants of smouldering wood and fields of hot ashes. Copulations often can be observed close to the various hot spots and after mating females deposit the eggs under the bark of burnt trees [4]. The larvae develop in the fire-killed trees and the beetles of the next generation start to hatch in the summer of the next year. Although so far no systematic studies on the reproductive success of *Melanophila* beetles exist, there is strong evidence that beetles cannot reproduce successfully in unburnt trees [1].

For more than 100 years several mainly anecdotal publications have suggested that even very small fires are attractive to *Melanophila* beetles [2], [8], [9]. Accordingly it has been reported that bigger fires attract large numbers of beetles over great distances [3], [5], [10]. In this context it is interesting to note that *Melanophila* beetles are not only attracted by forest fires. Beetles have been found near a small plant for tar production where they aggregated on hot masonries, pipes and tanks [11]. Untold numbers *of Melanophila consputa* were attracted to a burning 750,000 barrel oil storage tank near Coalinga in California [12]. Because Coalinga is situated in an arid part of California, the next coniferous forests which most probably were the source for the beetles were 50 to 100 miles away. *M consputa* also has been observed at lumber yards and sugar mills, and in great numbers at sugar refineries congregating about the vats where the hot sugar syrup was stored [3]. Great numbers of beetles have been attracted by a large smelter plant where the next coniferous forest was about 50 miles away [6]. During a big football game in the Californian Memorial Stadium at Berkeley *Melanophila* beetles swarmed in sufficient numbers to plague the audience by biting people in the necks or hands. It was concluded that about 20.000 cigarettes attracted *M. consputa* and *M. acuminata* both of which breed in fire-scorched pines in the hills adjacent to the stadium [10]. *Melanophila consputa* and *M. occidentalis* swarmed in numbers of several thousand individuals about two cement plants in southern California where the beetles congregate near the kilns at high ambient temperatures. Beetles were especially numerous in the vicinity of the burning zone of the kilns. It is also of importance to note that at the first plant the next coniferous forest was 20 miles away, at the second facility the distance to the nearest forest was 40 miles [13].

This gives rise to two hitherto open questions: (i) which physical cues are used by the beetles for the remote detection of fires? A list of potential cues which theoretically can be used by a beetle is given in Table 1. (ii) From which distance can a fire be detected by a beetle? This second question is directly linked to the sensitivity of the receptors used for fire detection.

**Table 1:** Physical cues suitable for fire detection.

SOURCE	CUE
Hot surfaces of burning material	IR++
Open flames	VIS++ (mainly at night)IR+
Hot airspace above burning material (e. g. forest)*	IR+
Smoke plume	OLF++ (depending on wind direction)VIS++

Abbreviations: IR: infrared radiation; VIS: visible light; OLF: olfactory stimulus.

++ : strong cue;

+ : moderate cue.

* the hot airspace contains heated air, carbondioxide, carbonmonoxide, various hydrocarbons, water vapour, nitric oxides, soot, tar, unburned particles, fine dust.

**Figure 1:**
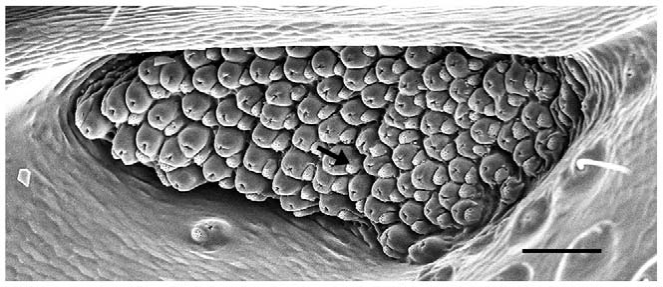
Right IR organ of *Melanophila acuminata* (head is up). At the bottom of a small pit about 70 IR sensilla can be found. Each of the dome-shaped sensilla is associated by small wax gland (see asterisk, bar 50 µm).

In two of the above reports no open flames or significant smoke plumes have been reported [3], [13]. The visible light of the flames can probably also be eliminated as an important cue for remote fire detection since *Melanophila* beetles are diurnal [10], [14]. Thus Table 1 suggests that infrared (IR) radiation may be an adequate cue for the remote detection of various high temperature events including forest fires. An indeed, a pair of sensory pit organ was found on the thorax on all the species of the *acuminata* or “flattened type” which fly to fires (i.e.: *M. consputa, M. notata, M. opaca, M. atropurpurea*, and *M. acuminata*). The pits are contiguous with the lateral margin of the coxal cavities of the middle pair of legs [15]. Later on it could be shown by behavioural [16], [17] and also by electrophysiological experiments [18], [19] that these pit organs are IR receptors. At a first glance the receptor organ is reminiscent of a small complex eye because it is composed of about 70 dome-shaped IR sensilla building a small sensor array (Fig. 1). This complex construction suggests that the *Melanophila* IR organ may serve for demanding sensory purposes. Furthermore, morphological investigations revealed that the infrared sensilla inside the pit organ are innervated by ciliary mechanoreceptors [20], [21] which belong to the most sensitive receptors in the animal kingdom [22], [23].

In the present paper we want to investigate from which distances a fire can be detected by the IR organs. In this way we want to get new insight about the absolute sensitivity of the IR receptors. Sensitivity thresholds have been determined by the above mentioned behavioural (0.6–1 W/m^2^; Evans [16]) and electrophysiological experiments (lowest threshold of 5 W/m^2^ published by Schmitz and Bleckmann [18]). However, due to methodical reasons it is doubtful whether these data represent the real thresholds (see Discussion for more details). By using these measured thresholds, Evans and Schmitz and Bleckmann calculated from which distances *Melanophila* beetles should be able to detect hypothetical fires with an assumed size between 10 and 20 hectare (i.e. 100–200.000 m^2^). Distances do not exceed 12 km which is far below the distances mentioned in several of the older reports.

In the current attempt we assume that *Melanophila* beetles use IR radiation for remote fire and heat detection. We made use of the physical and thermal conditions of the big oil tank fire in Coalinga which attracted enormous numbers of *Melanophila* beetles [12]. As mentioned, nearest forests were at least 50 miles (80 km) away. Because IR emission of a burning oil tank can be calculated much better than emission of a fast spreading forest fire, we are sure that the infrared source could be defined with sufficient accuracy. Based on the results of our modelling we could show that it may be possible that *Melanophila* beetles could detect large fires by IR radiation from distances up to 130 km.

## Methods

To estimate the sensitivity of an IR sensillum, a model is required that comprises the IR emission of a fire source as a function of distance. As a basic precondition it has to be postulated that the beetles had started their flight at a distance where the calculated irradiation intensity was at least slightly larger than the threshold of the sensillum. So it is necessary to have sufficient data about the fire and to know the distance between the habitat of the beetles and the fire. The report of van Dyke offers only little information about the oil tank fire and the appearance of *Melanophila* beetles attracted by the fire:

1. The fire source was a 750,000 barrel storage of Shell Oil Company.
2. The fire took place during August 1925 at Coalinga, California.
3. It is supposed that the flight distances of the beetles were 50 to 100 miles because Coalinga is situated in the arid Central Valley of California where the next forests can be found in western or eastern direction at distances of 50 or 100 miles respectively.

Based on the historical report we started to retrieve more data from various sources.

### Identification of the fire place and fire data

Diana Baker of the Coalinga Huron Library, Coalinga, helped us to get access to the archive of the newspaper “Coalinga Daily Records”. It was reported that the outbreak of the fire was on August 10^th^, 1925, due to a lightning strike at 11:20 in the morning, 9 miles east of Coalinga on section 36. In the evening the burning tank, containing high gravity refining oil, boiled over, ignited the rest of the reservoir and converted the little valley where the reservoir was located in “a lake of fire”. The flame height was “hundreds of feet” within “a huge curtain of smoke”. The greatest flash “shot over 500 feet into the air and was visible for more than 30 miles”, “the light of the fire was so great that one could easily read by it in town, a distance of nine miles”. The tank “ is nested in a little canyon ….and entirely surrounded by hills except for one little opening northeast of the tank, which will let the burning oil drain out the plains …”. The fire went out not later than August 12^th^.

**Figure 2:**
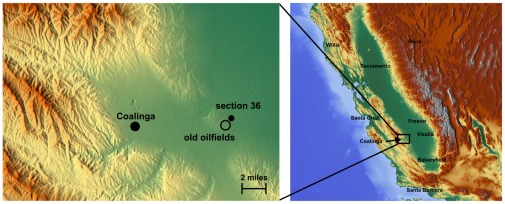
Identification of section 36 and the place of the old oilfields using [**24]**, [98****].

For the identification of the fire place the information of the section 36 was very valuable. In United States the Public Land Survey System (PLSS) is used to divide land into 6-mile-square townships, which is subdivided into 36 one-mile- square sections. Using a software [24] it was possible to map the Public Land Survey System onto Google Earth and to locate section 36, see Fig. 2. The place of the fire in the year 1925 must be somewhere in the hills west of the marker “section 36”. The reported distance to Coalinga is correct, but the little canyon where the fire took place could not be identified free of doubt due to the low resolution of the maps.

### Identification of the sources of beetles and of the potential flight routes

As pointed out in detail in the first section of the Discussion, the beetles observed at the tank fire most probably mainly originated from two areas: At a distance of about 16 miles the southern edges of the tree covered area around forest of the San Benito Mountain Natural Area is located (in the following called “San Benito Mountain”). In a much larger distance of about 80 miles the western foothills of the extended forests of the Sierra Nevada are located. Here two larger forest fires were identified in 1923 (Muir Grove) and 1924 (Kaweah River). As outlined in the Discussion, beetles which stayed on the wooded western slopes of the Sierra Nevada (in the following called “foothills of the Sierra Nevada”) had a good chance to become aware of the fire.

### Fire models

For the fire safety and risk analysis of buildings or technical equipment theoretical fire scenarios have been established termed “pool fires”. In a pool fire simulation a burning liquid fuel in a gas or oil storage tank spreads flames over the horizontal fuel surface. By means of the quantitative methods developed in “Fire Dynamic Tools” [25] and “SFPE Handbook of Fire Protection Engineering” [26] fire protection inspectors are able to perform risk-orientated evaluations of fires which can produce a high heat load due to a thermal radiation field. The fire models are based in part on empirical calculations derived from real fires from past incidents or fire experiments or analytical methods. This proven knowledge cannot only be used to calculate the heat load on nearby buildings but also for the determination of the radiation flux at an arbitrary spot far away from the fire. For the calculation of the radiation flux three models were established: the point source model, the conventional solid flame model and a modified solid flame model [25], [26], [27], [28].

#### Point source model

**Figure 3:**
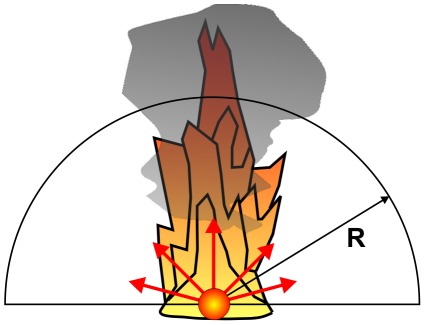
Point source model.

The “point source model” is very simple. It is assumed that a fraction of the total fire energy is released as thermal radiation. Thermal radiation is emitted by a point source representing the flame and the radiated energy is distributed over a surface area of a sphere whose radius x is the distance from the fire to the target [25], [26], [27], [28], see Fig. 3. The point source model overestimates the heat flux near to the fire and should not be used for distances x smaller than some diameters of the fire. Therefore, at closer distances the assumption of a point source is not valid [28].
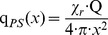
(1)with: q_PS_(x) [W/m^2^]: radiant heat flux at the target in distance x, Q [kW]: heat release rate of the fire, χ_r_: fraction of the total fire energy released.

The fraction of the total fire energy released, χ_r_, depends on the fuel, the flame size and the flame configuration and varies from approximately 0.15 to 0.6, depending on the sooting of the fuel [25]. For hydrocarbon fuels χ_r_ is correlated with the fire diameter D, based on fire experiments with pool diameters D smaller than about 40–50 m [28]:
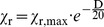
(2)with: D [m]: pool diameter, D<40 m–50 m, χ_r,max_ = 0.35

Equation (2) shows that χ_r_ decreases strongly with the pool diameter D; in [28] it is proposed that χ_r_ remains stable for large diameters, e. g. χ_r_ = 0.07–0.1. For crude oil χ_r_ = 0.1–0.02 for fire diameters between 5 and 30 m.

In [26] an alternative relation for χ_r_ is proposed:
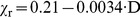
(3)with: D [m]: pool diameter, D<50 m

For pool diameters of more than 50 m values of χ_r_ for 50 m, 0.03–0.06, should be used. For a pool diameter of 20 m and a target distance of x = 1,000 m, Equation (2) yields χ_r_ = 0.13 and Equation (3) yields χ_r_ = 0.14. In this case (Q = 60 MW) the radiant heat flux is 0.6–0.7 W/m^2^ at a distance of 1,000 m.

The heat release rate of the fire, Q, can be calculated with [29]:
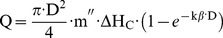
(4)with: m″ [kg/m^2^⋅s]: mass burning rate per area, ΔH_C_ [KJ/kg]: lower heat of combustion of the burning fuel, kβ [1/m]: empirical constant. For crude oil [29] values are: m″ = 0.0335 [kg/m^2^⋅s], ΔH_C_ = 42,600 [KJ/kg], kβ = 2.8 [1/m].

#### Solid Flame Model and modified Solid Flame Model

**Figure 4:**
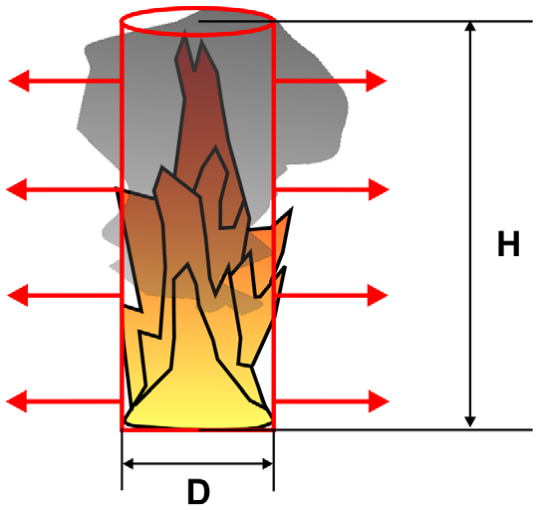
Solid flame model.

The “solid flame model” assumes that the fire can be represented by a simple geometrical shaped body, in most cases a cylinder, wherein the thermal radiation is emitted from its surface [25], [26], [28], [30], see Fig. 4. The radiant heat flux is calculated as:
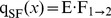
(5)with: q_SF_(x) [W/m^2^]: radiant heat flux at the target in distance x, E [kW/m^2^]: average emissive power at the flame (cylinder) surface, F_1→2_: configuration factor or geometrical view factor between the radiating surface and the surface of the object.

### Emissive power

The calculation of the emissive power E is the critical part when using the solid flame model. For the calculation of the emissive power different correlations can be used. All equations are based on experimental data. For that correlation the flame is assumed to be a uniformly radiating cylindrical black body. The first equation is given by [29]:
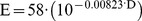
(6)with: D [m]: pool diameter, D<60 m

Especially for large pool diameters an alternative equation is proposed by [31]:
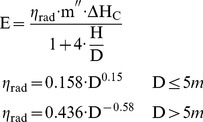
(7)with: H [m]: flame height, m″ [kg/m^2^⋅s]: mass burning rate per unit area, D<50 m

For η_rad_ also χ_r_ from Equation (2) can be used [31]. Equation (5) assumes a constant emissive power over the whole surface of the cylindrical flame. Due to the black smoke causing a reduction in radiation at the upper part of the flames – the emissive power actually is not constant over the entire surface. This is especially true if the pool diameter increases. Equation (6) takes account for this fact with a correction factor depending on the pool diameter [26].

**Figure 5:**
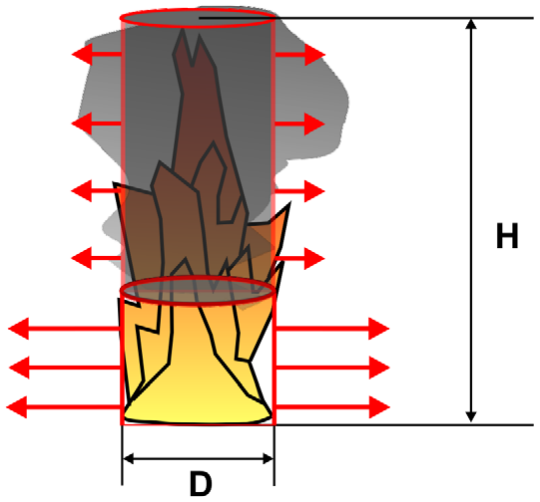
Modified Solid flame model.

Actually the flame is divided into two parts: a luminous part where the flames can be clearly seen with high emissive power and an upper larger part where dark smoke covers the flame with sudden bursts of luminous flames. Here the emissive power is reduced due to the smoke. The moving border between these two parts depends on fuel, pool diameter, and oxygen content of the burning zone etc. [32]. For a flame idealized as a cylinder this means that on average about 20% of the surface of the cylinder consists of visible flames with high heat radiation and 80% is smoke with lower heat radiation [28]. The modified solid flame model, see Fig. 5, take this into account [31].

A modified equation for the emissive power of a two zone modified solid flame was developed by [31]:
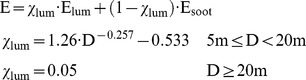
(8)with: E_lum_ = 115 kW/m^2^, E_soot_ = 40 kW/m^2^, all values being for diesel oil

A similar equation is given in [26]:
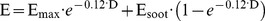
(9)with: E_max_ = 140 kW/m^2^ (equivalent blackbody emissive power), E_soot_ = 20 kW/m^2^

Assuming the modified solid flame model and a pool diameter of 50 m Equation (9) yields an emissive power of 20 kW/m^2^ compared to 44 kW/m^2^ using Equation (8). For the same pool diameter the solid flame model yields an emissive power of 38 kW/m^2^ using Equation (6) and about 16–17 kW/m^2^ using Equation (7). The ranges of the emissive power calculated with the solid flame model and the modified solid flame model are comparable. However, a factor of about two must be accepted between the higher and lower values.

### Flame height

Because the real fire is replaced by a radiating cylinder, the calculation of the height H of the cylinder is an important issue. Here some different relationships exist which are based on experimental data. Based on laboratory-scale fires in absence of wind the following equation is given by [33]:
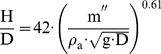
(10)With: H [m]: flame height, ρ_a_ [kg/m^3^]: ambient air density, m″ [kg/m^2^⋅s]: mass burning rate per unit area, g: acceleration of gravity, g = 9.806 m/s^2^

For pool fires with large diameters and many liquid fuels an alternative equation was developed [34]:
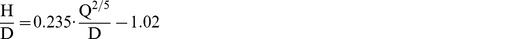
(11)With: Q [kW] heat release rate of the fire, see Equation (4).

For a pool fire of crude oil with a diameter of 50 m Equation (10) yields a flame height of 34 m and, with an appropriate heat release rate of the fire Q = 2.8⋅10^6^ kW, Equation (11) results in a flame height of 38 m. Both values are consistent when the empirical basis of the equations is taken into account.

### View factor

The view factor F_1→2_ describes the fraction of radiation energy diffusely emitted by object 1 that arrives at the area of object 2.

**Figure 6:**
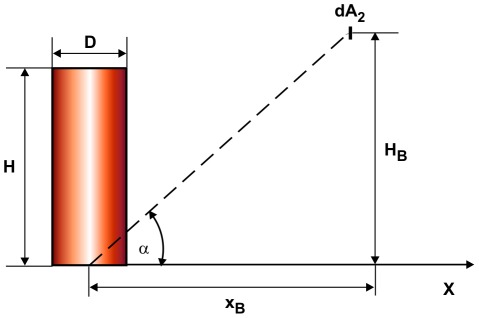
Calculation of the view factor.

For the analysis a view factor is necessary that considers the flying altitude of the beetle, H_B_, and the distance to the flame, x_B_, see Fig. 6. In [35] an appropriate view factor was presented, which also considers the inclination angle of the cylinder (flame) axis, e.g. due to wind. Here this angle will be neglected, because the slight reduction of the emitted energy is less than the uncertainty of the calculation of the flame height. The view factor F_1→2_ from [35], simplified by neglecting any inclination angle of the cylinder is
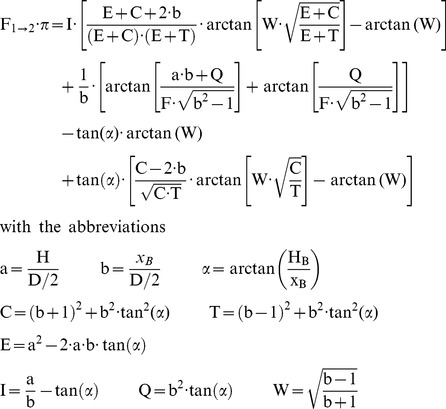
(12)


With: H: height of cylinder (flame height), D: diameter of cylinder (pool diameter), H_B_: flying altitude of the beetle, x_B_: distance of the beetle to the flame, see Fig. 6.

**Figure 7:**
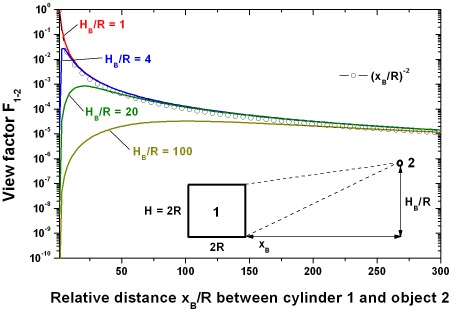
View factor F_1→2_ between object 1 (cylinder, flame) and object 2 (beetle) as a function of the relative distance. The height of the cylinder is equal to the diameter and the flying altitude of the beetle, H_B_, is normalized to the radius R of the cylinder. For large relative distances x_B_/R F_1→2_ can be reduced to (x_B_/R)^−2^.

Fig. 7 shows the view factor F_1→2_ as a function of the relative distance x_B_/R and the relative flying altitude of the beetle H_B_/R with R as radius of the pool fire. When the altitude is lower than the height of the cylinder the view factor reaches its maximum value 1. However, when the altitude is higher than the height of the cylinder, the maximum cannot be reached because close to the cylinder only a small projection of the lateral area of the cylinder is visible and the top of the cylinder is supposed as non-radiating. It is obvious that the altitude of flight has only an influence on the view factor in the vicinity of the cylinder; in the far distance this influence can be neglected. Consequently, far away from the fire the knowledge of the altitude of flight is less important. For large relative distances x_B_/R the view factor F_1→2_ can be reduced to (x_B_/R)^−2^.

### Atmospheric absorption and scattering

The radiant heat flux between a flame and an object normally will be reduced due to absorption in the atmosphere and attenuated by airborne particles like dust, smoke and fog. The absorption is mostly caused by water vapour and carbon dioxide. The reduced radiant heat flux can be calculated with:
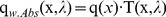
(13)with: q_w.Abs_(x,λ) [W/m^2^]: radiant heat flux at the target at a distance x from the source with absorption; q(x) [W/m^2^]: radiant heat flux at the target in distance x without absorption due to Equation (1) or (5), T(x,λ) [-]: transmittance

The transmittance T of the radiant heat flux is described by the Lambert–Beer law as a function of the wavelength λ:
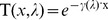
(14)with: γ(λ) [1/km]: extinction coefficient

The extinction coefficient γ(λ) includes an absorption and a scattering term:
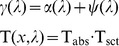
(15)with: α(λ) [1/km]: molecular and aerosol absorption coefficient, ψ(λ) [1/km]: molecular and aerosol scattering coefficient, T_abs_: transmissivity due to molecular and aerosol absorption, T_sct_: transmissivity due to molecular and aerosol scattering

The mean extinction coefficients of the atmosphere for a wavelength window mentioned in the literature very often differ significantly because the measurement depends on the detector, the spectral interval, exact atmospheric conditions, path length etc.. Therefore different calculation methods will be compared.

An important question is the bandwidth of the radiation of a pool fire and the bandwidth of the absorption by the sensillum of the beetle. The emission spectra of a pool fire larger than a few meters can be described by a black body radiator with a temperature Θ_s_ of about 1,300 K [26] or 1,500 K [36]. For a black body radiator with the temperature Θ_s_ of the radiating surface the spectral distribution of the emissive power into a gas with refraction close to unity can be described by Planck's law [37]

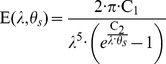
(16)With: E(λ, Θ_s_) [W/m^3^]: hemispherical spectral emissive power, Θ_s_ [K]: temperature of the radiating surface, C_1_ = 5.9552197⋅10^−17^ Wm^2^, C_2_ = 1.438769⋅10^−2^ m⋅K

The fraction of emissive power emitted into a given wavelength window between λ_1_ and λ_2_ is [37]:
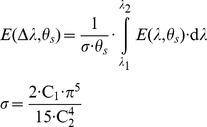
(17)With: Δλ = λ_2_–λ_1_ [m]: wavelength window, σ = 5.67051⋅10^−8^ [W/(m^2^ K^4^)]: Stefan- Boltzman constant

**Figure 8:**
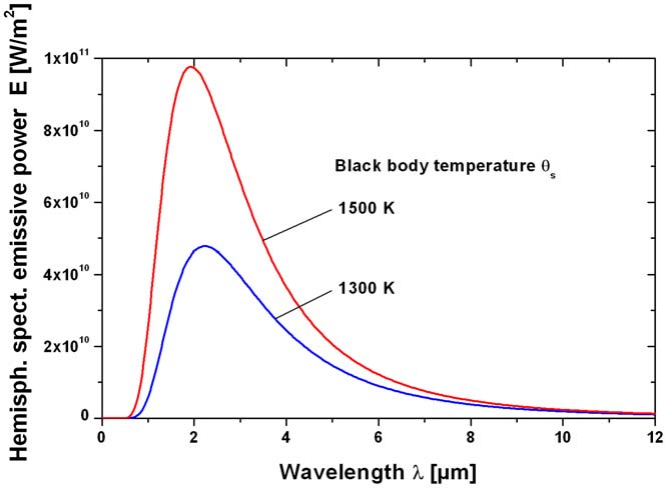
Hemispherical spectral emissive power E of a black body radiator with a surface temperature of Θ_s_ = 1300 K and 1500 K as function of wavelength.

Fig. 8 shows the hemispherical spectral emissive power as function of the wavelength and the radiating temperatures 1,300 K and 1,500 K. The maximum radiation occurs at a wavelength of about 2 µm with smaller radiation portions of up to 12 µm. A limitation of the bandwidth between e.g. 3–5 µm as in IR technical sensors (atmospheric window) would only capture 27% (Θ_s_ = 1,500 K) or 31% (Θ_s_ = 1,300 K) of the available radiating power using Equation (17). Most probably, however, the biological IR sensor will use the full bandwidth to reach the maximum sensitivity.

**Figure 9:**
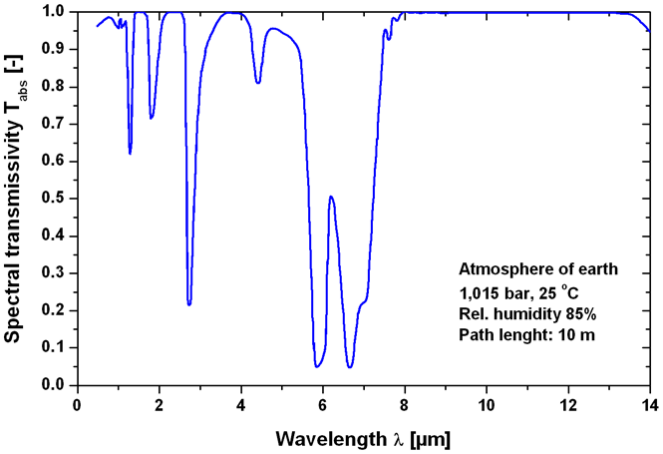
Atmospheric spectral transmissivity at a path length of 10 m. Air temperature 25°C, air pressure 1.015 bar, relative humidity 85%. Based on a diagram in [39].

The spectral transmissivity is the radiant flux which passes through a filter divided by the radiant flux incident upon it, for monochromatic light of a specified wavelength [38]. The spectral transmissivity of the atmosphere (temperature 25°C, air pressure 1,015 bar, relative humidity 85%) is shown in Fig. 9 with a path length of 10 m using data from Walther [39]. The comparison of the spectral emissive power in Fig. 8 with the spectral transmissivity in Fig. 9 yields an effective range between 1–5 µm where the radiation of a fire can pass a wavelength window with low transmission loss.

A determination of the average transmittance in 8 windows between λ_1_ = 0.72 and λ_2_ = 15 µm was presented by [36]. This formula assumes equal amounts of radiant power at all wavelengths, which is strictly speaking not true for a pool fire as black body radiator of 1,500 K. The formula allows choosing all windows or a partition regarding the examined wavelength range. The average transmittance due to molecular absorption is [36]:
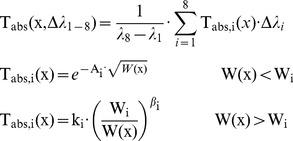
(18)with: T_i_(x) [-]: Transmittance in window i, wavelength window: λ_1_ = 0.72 to λ_2_ = 15 µm W(x) [mm]: Precipitable millimetres of water in the path length x; W_i_, A_i_, k_i_, β_i_: Constants defined below

The constants W_i_, A_i_, k_i_ in Equation (18) depend on the wavelength window, see Table 2.

**Table 2:** Constants used in Equation (18) [36].

window i	Δλ_i_	λ_i_	W_i_	Ai	ki	β_i_
1	0.72–0.94 µm	0.83	54	0.0305	0.800	0.112
2	0.94–1.13 µm	1.04	54	0.0363	0.765	0.134
3	1.13–1.38 µm	1.26	2.0	0.1303	0.830	0.093
4	1.38–1.90 µm	1.64	1.1	0.211	0.802	0.111
5	1.90–2.70 µm	2.30	0.35	0.350	0.814	0.1035
6	2.70–4.30 µm	3.50	0.26	0.373	0.827	0.095
7	4.30–6.0 µm	5.15	0.18	0.913	0.679	0.194
8	6.0–15.0 µm	10.5	0.165	0.598	0.784	0.122

The precipitable water W(x) is a parameter often used in metrology. It means that the whole water vapour content in a column of unit-cross sectional area and path length x is condensed and at the end of the column a height W(x) exists, measured in mm, finally resulting in the same absorption compared to the common distributed water vapour in the column. Obviously W(x) depends on the relative humidity, the air temperature, the saturated vapour pressure at the air temperature, and the length of the column (path length)
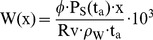
(19)with: ϕ [-]: Relative humidity, P_s_(t_a_) [Pa]: Saturated vapour pressure at the atmospheric temperature t_a_ [K], Rv = 461.5 Nm/(kg⋅K): Gas constant of water vapour, ρ_w_ = 998 kg/m^3^: density of water

For a relative humidity of 0.85 (Fresno, Joaquin valley, average value for August in the early morning hours), air temperature 25°C (P_s_ = 3.2 Kpa) and a path length of 10 m a precipitable water content of 0.198 mm results. Equation (18) yields a transmittance of 0.726 and using Lambert – Beer law, Equation (14), a molecular absorption coefficient α_Kruse_ = 32 1/km (6.0–2.7 µm). For a path length of 100 m results α_Kruse_ = 6.05 1/km.

In [40] a formula was proposed for calculating the atmospheric infrared transmissivities for a wavelength window between 1–18 µm. The formula is valid for path lengths between 10–1000 m at air temperatures between 253–313 K. A pool fire as a black body radiator at 1,500 K is assumed.
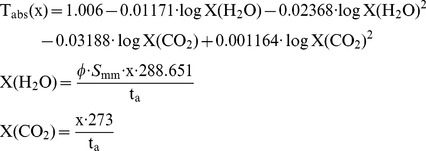
(20)with: ϕ [-]: Relative humidity, S_mm_: Saturated water vapour pressure in mm mercury at the atmospheric temperature t_a_ [K]. If ϕ = 0 then X(H_2_O) = 1

For the same atmospheric conditions as above (relative humidity of 0.85, air temperature 25°C) Equation (20) together with the Lambert – Beer laws, Equation (14), yields a molecular absorption coefficient α_Wayne_ = 19.3 1/km for a path length of 10 m and 43.0 1/km for a path length of 100 m. Both Equations (18) and (20) yield an absorption coefficient which depends on the path length.

**Figure 10:**
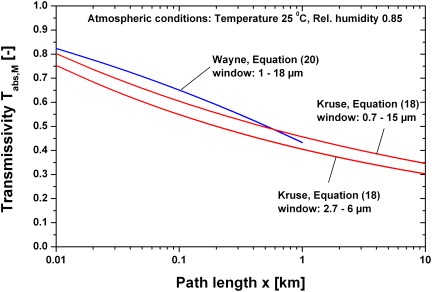
Comparison of atmospheric transmissivities (25°C, relative humidity 0.85, different wavelength windows) due to molecular absorbance. The mean transmissivity T_abs_,_M_ was calculated in the mentioned wavelength window.

Fig. 10 shows a comparison of the atmospheric transmissivities using the Equations (18) and (20) as function of the path length. In the range of validity the transmissivities based on Equations (18) and (20) show a satisfying conformance. Equation (18) will be used for further calculations because in equation (20) the path length is limited to 1 km and the wavelength window is too large.

The calculation of the damping of the radiant heat flux due to scattering is based on different physical phenomena. In the field of Free Space Optical communications (FSO) where data are transmitted by optical or infrared signals the damping of the signal is a critical factor. In FSO the visibility or visual range V characterizes the transparency of the atmosphere as a quantity estimated by a human observer. It can be measured by the Runway Visual Range (RVP) which is defined as the distance which parallel light at a colour temperature of 2,700 K (yellow/green, λ = 0.55 µm) must travel so that its intensity is reduced to 5% or 2% of its original value. For very clear air the visibility is 50–20 km, for clear air 20–10 km and for light mist 2–4 km [41].

In [41] an equation is proposed to calculate the transmissivity at the midpoint of the same wavelength windows used in Equation (18).
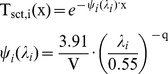
(21)with: V [km]: Visibility, λ_i_ [µm]: Midpoint of i_th_ wave length window i, Table 2

**Figure 11:**
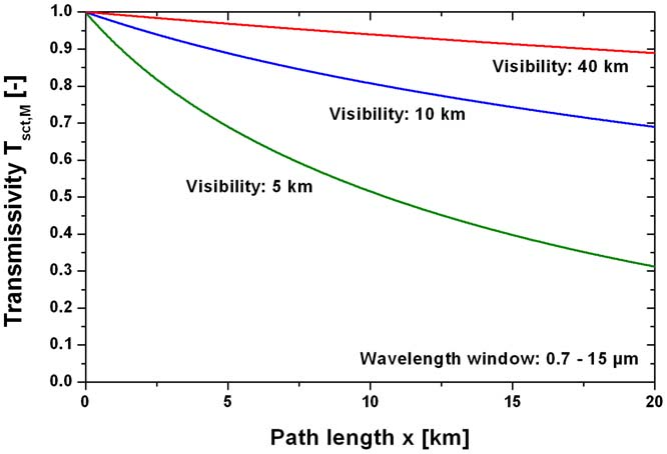
Atmospheric transmissivity due to scattering for different visibilities. The mean transmissivity T_sct_,_M_ was calculated in the wavelength window 0.7–15 µm.

The coefficient q depends on the visibility V is [36], [42], [43]

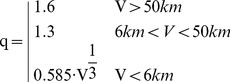
(22)The transmissivities using Equations (21) and (22) are shown in Fig. 11 for the visibilities 5, 10 and 40 km. It is obvious that a visibility of only some km reduces the signal significantly.

**Figure 12:**
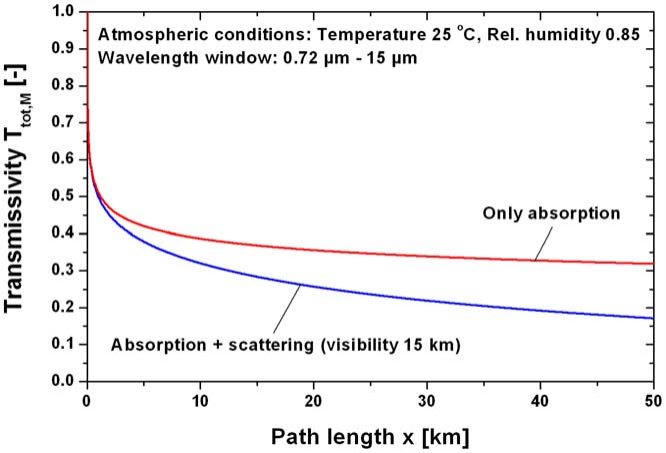
Comparison of atmospheric transmissivity with and without scattering due to a visibility of 15 km. The mean transmissivity T_tot_,_M_ was calculated in the wavelength window 0.7–15 µm.

Concurrently, occurring transmission losses due to absorption and scattering can be calculated with a combination of the Equations (18) und (21) [36]

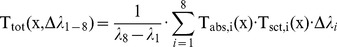
(23)
Fig. 12 shows a comparison of the transmissivity with and without absorption/scattering (visibility 15 km). At a distance of 50 km the transmissivity due to absorption is 0.31, additional scattering reduces the transmissivity to 0.17.

### Comparison of the two fire models

**Figure 13:**
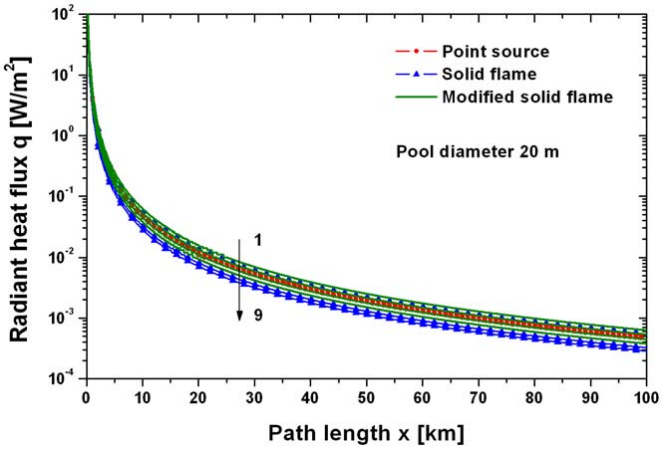
Comparison of the point source model, solid flame model and modified solid flame model for a crude oil pool fire with a diameter of 20 m without loss due to absorption and scattering. The numbers indicate: 1 modified solid flame model, emissive power Muñoz, Equation (8), flame height Heskestad, Equation (11). 2 solid flame model, emissive power FDT, Equation (6). 3 modified solid flame model, emissive power Muñoz, Equation (8), flame height Thomas, Equation (10). 4 point source model, emissive power Beyler, Equation (3). 5 point source model, emissive power McGrattan, Equation (2). 6 modified solid flame model, emissive power Beyler, Equation (9), flame height Heskestad, Equation (11). 7 modified solid flame model, emissive power Beyler, Equation (9), flame height Thomas, Equation (10). 8 solid flame model, emissive power Muñoz, Equation (7), flame height Thomas, Equation (10). 9 solid flame model, emissive power Muñoz, Equation (7), flame height Heskestad, Equation (11).

Fig. 13 shows the radiant heat flux for a pool fire with a diameter of 20 m without loss due to absorption and scattering as function of the target distance for the point source model and the solid flame model. Influence of the different approaches to calculate the emissive power and flame geometry are included. Obviously the point source model, the solid flame model and the modified solid flame model are in good agreement for larger path lengths x (x≫D). Even the simple point source model is a correct assumption if distances between the fire and target are large [26].

**Figure 14:**
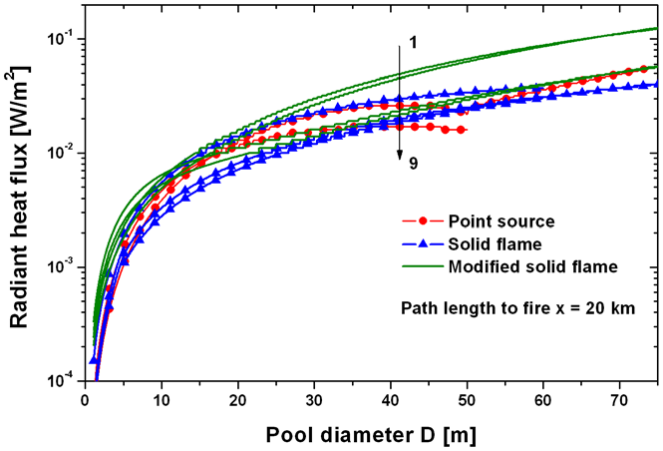
Comparison of point source model and modified solid flame model with pool diameter as parameter without loss due to absorption and scattering. 1 modified solid flame model, emissive power Muñoz, Equation (8), flame height Thomas, Equation (10). 2 modified solid flame model, emissive power Muñoz, Equation (8), flame height Heskestad, Equation (11). 3 solid flame model, emissive power FDT, Equation (6). 4 point source model, emissive power Beyler, Equation (3). 5 modified solid flame model, emissive power Beyler, Equation (9), flame height Heskestad, Equation (11). 6 modified solid flame model, emissive power Beyler, Equation (9), flame height Thomas, Equation (10) (11). 7 solid flame model, emissive power Muñoz, Equation (8), flame height Heskestad, Equation (11). 8 solid flame model, emissive power Muñoz, Equation (8), flame height Thomas, Equation (10). 9 point source model, emissive power McGrattan, Equation (2).

Fig. 14 shows, again without loss due to absorption and scattering, the radiant heat flux as function of the pool diameter for the different flame models. Here the radiant heat flux increases considerably until a pool diameter of 20–30 m has been reached. All models except the solid flame model using the emissive power by Equation (8) predict a comparable slightly increasing radiant heat flux for pool diameters larger than about 40 m. This means that the exact knowledge of the pool diameter in the third phase of an oil fire, see section “Geometry of the pool fire”, is not so important.

A comparison of the results of the point source model, the solid flame model and the modified solid flame model for a Toluene pool fire with a diameter of 30 m [26] yields a 20% higher radiant heat flux of the point source model versus the solid flame model and the modified solid flame model. It is recommended to use the point source model only for an emissive power of the fire smaller than 5 kW/m^2^ [26].

In order to test the prediction of the different models, real pool fires with measured heat fluxes were compared with the calculated heat fluxes. For a measured heat flux of e.g. 3–4 kW/m^2^ the calculated values of the point source model showed a good agreement within a range of ±20% of the calculated values. For higher heat fluxes the point source model underestimates the measured heat fluxes by a factor of about 2 [26]. A calculation of the measured heat flux up to 5 kW/m^2^ with the solid flame model showed a good agreement within a range of ±20% of the calculated values, for values up to 15 kW/m^2^ for the measured heat flux the calculated values underestimates the measured values up to 20% [26]. This means that the fire models underpredict the heat fluxes at closer locations, for larger distances especially the solid flame model is in good agreement with the measured heat fluxes. Therefore it is recommended in [26] to use no safety factor for realistic results.

Overall, the presented fire models are very suitable for this study and results in realistic predictions of the heat fluxes at distances where the beetles most probably had started their flights towards the fire.

### Input data for the calculations

#### Weather data in Coalinga, August 1925

The knowledge of temperature, humidity and visibility is important for the calculation of the atmospheric absorption and scattering. As it is not possible to find out the real weather conditions of August 10^th^, 1925, long-time averaged data for this region are used.

The average temperature for August (1942–2005) in Coalinga is 36°C (max) and 17°C (min). The mean temperature at August 10^th^ is 27°C [44]. The average relative humidity for August in Fresno, 80 km away from Coalinga in the Joaquin Valley, is 67% (morning) and 25% (evening) [45]. August is a very dry month with only 0.1 mm average rainfall (1931–1951) in Coalinga [46]. Documented values for the visibility in Coalinga in the twenties were not found. In August on average 26 days are clear in Coalinga, only 4 days are cloudy or partly cloudy [47]. For 1950 and later the historical visibility trends were reported [48]. Here the mean visibility (3^rd^ quarter of the year) in the San Joaquin Valley descends almost linearly from about 20 miles in 1950 to 13 miles in 1965. Based on this trend and that the days in August are mostly clear a high visibility of 20–30 miles in August 1925 in Coalinga is a realistic assumption. The newspaper “Coalinga Daily Records” reported August 10^th^, 1925, that the greatest fire flash from the oil tanks “shot over 500 feet into the air and was visible for more than 30 miles”. This observation confirms our assumption.

The wind direction and the wind speed are also important parameters. If the wind would blow the fire plume against the flight route, than the fire plume can reduce the visibility. Actually the main wind direction is about crossways to flight route because during summer months, the predominant surface wind direction in the San Joaquin Valley is from the northwest to southeast, down valley from Stockton towards Bakersfield. Wind speed increases during the day, shifting towards a northwest to southeast direction, and peaking around 5:00 p.m. Pacific Daylight Time [49]. The average wind speed in August (1996–2006) at Fresno was 7.2 mph, with annual variations from 3.9 to 9.1 mph [50]. In Bakersfield at the lower part of the San Joaquin Valley the wind speeds have only very slight variations from the wind speeds in Fresno. This means that these wind speeds are representative for the San Joaquin Valley.

According to [26] a wind speed below 1.72 m/s (pool diameter 20 m) will not tilt the flame axis, a wind speed of 3.2 m/s will reduce the flame height by about 10%. Compared to the insecurity of other input data the reduction of the flame height will be neglected.

#### Geometry of the pool fire

**Figure 15:**
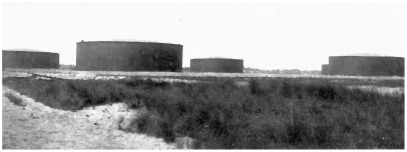
55.000-barrel storage tanks from Mexican Eagle Oil Co., beginning of the 20^th^ century [**99**].

Despite intensive efforts to identify the exact number and geometry of the tanks of the Coalinga reservoir no source was found that describes the reservoir in detail. In spite of this, the geometry of the tanks can be determined almost certainly. In the early 20^th^ century 35.000–55.000-barrel steel tanks were commonly used in large storage areas [51]. The diameter of a 35.000 - barrel tank was about 80–90 feet (24–27 m) [52] and the diameter of a 55.000 - barrel tank was 114.5 feet (35 m) [53]. The reservoir may have looked similar to the tank reservoir consisting of 55.000-barrel tanks, see Fig. 15.

The geometry of the pool fire changes in the course of burning time. During the first phase the fire pool diameter is identical with the diameter of the tank. This first phase lasts about 6–7 hours. During this time the tank and its oil content is continuously heated up. Due to condensation effects, drilling and transport or the natural composition of the oil water is present in the tank, which is located as a sediment layer in the bottom of the tank. When this layer reaches the boiling temperature it starts to vaporize and the fuel is ejected from the water steam [54].

Because of a superheating of the water and the hydrostatic pressure of the oil above the water layer this happens suddenly and can be compared with an enormous explosion. In this second phase the flame height and the emissive power both increased considerably. The “Coalinga Daily Records” from August 11^th^ described the boil over with “the flames shot over 500 feet into the air” at 6:30 p.m.. The duration of this second phase is not reported, but from other accidents it can be assumed that this takes about some minutes until the water steam is released from the tank. A boil over can happen several times depending on the water volume in the tank.

Due to the boil over a lot of oil was ejected from the tank and covered the ground as a burning oil layer. The “Coalinga Daily Records” from August 11^th^ reported that the little valley where the oil storage was located changed into a lake of fire. This third phase did not last longer than August 12^th^.

The diameter of the pool fire and the flame height for the three phases as input for the radiant heat flux calculations is shown in Table 3. Most probably the pool diameter for the phase 3 was larger than 50 m, because of limitations of the empirical formulas the diameter is set to the maximum scope. This restriction can be tolerated because the radiant heat flux of large pool diameters increases only slowly for diameters larger than about 40 m, see Figure 13.

**Table 3:** The diameter of the pool fire and the flame height as input for the heat flux calculations for the three phases of the Coalinga oil fire.

phase	duration	pool diameter [m]	flame height [m]
1	∼6–7 h	24–35	Equation (19), (11)
2	∼0.5–1 h	24–35	150 m
3	∼36–48 h	50 m	Equation (19), (11)

## Results

### Radiant heat flux at the presumed sources of the beetles

As already mentioned in section “Identification of the sources of beetles and potential flight routes” and extensively discussed in section “Source of the beetles” of the discussion, the beetles most probably originate from regions with 16 miles (San Benito Mountain) or 80 miles (forests covering the western foothills of the Sierra Nevada) distance to Coalinga. For the calculation of the radiant heat flux during the three phases of the fire the data presented in Table 3 are used. For phase 1 and 2 where only one burning tank is assumed, the diameter is set to a low diameter of 30 m because it could not be identified if the tank size in Coalinga was 55.000 barrels (diameter 35 m) or 35.000 barrels (24–27 m). In phase 3 where the blaze turned into a lake of fire due to boil over of the tank, the diameter of the pool fire was set to 50 m because some of the equations used in our modeling are limited to this value. The visibility was set to 25 miles in phase 1 and 3 because of the estimations presented in section “Input data for the calculations”. Only in phase 3 the reported value of 30 miles was used. For the ambient temperature the reported mean value of 27°C in August in Coalinga was used. The relative humidity was chosen with respect to the daytime of the different phases, that means a higher humidity for morning hours (phase 1 and 3) and lower values for evening hours (phase 2).

**Figure 16:**
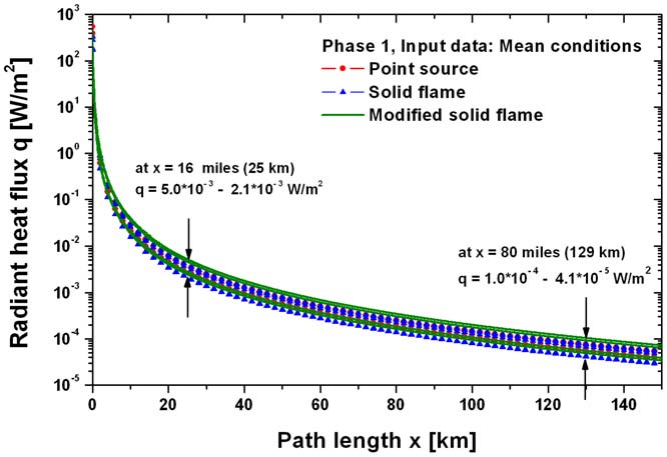
Radiant heat flux in phase 1 as function of the path length x based on the parameters in **Table 3** **(mean conditions, phase 1 of the fire).** For the distance of 16 miles between the Coalinga fire and the San Benito Mountain a radiant heat flux of 5.0⋅10^−3^ to 2.1⋅10^−3^ W/m^2^ results using the 9 different fire models mentioned in Fig. 13 and 14. The distance of 80 miles between the Coalinga fire and the foothills of the Sierra Nevada yields a radiant heat flux of 1.3⋅10^−4^ to 4.1⋅10^−5^ W/m^2^.

Based on the parameters in Table 4 the radiant heat fluxes shown in Fig. 16 were calculated for the phase 1 of the fire. In 16 miles distance to Coalinga at San Benito Mountain a radiant heat flux of 5.0⋅10^−3^ to 2.1⋅10^−3^ W/m^2^ results for phase 1 using the 9 different fire models depicted in Figs. 12 and 13. For the distance of 80 miles between the Coalinga fire and the starting point of the beetles at the western foothills of the Sierra Nevada a radiant heat flux of 1.3⋅10^−4^ to 4.1⋅10^−5^ W/m^2^ was calculated for phase 1.

**Table 4:** Parameters for the calculation with respect to the three phases P1, P2, P3 of the fire (mean conditions).

	Pool diameter D [m]			Visibility V [miles]			Air temperature T [°C]			Rel. humidity ϕ [%]		
	P1	P2	P3	P1	P2	P3	P1	P2	P3	P1	P2	P3
Mean	30	30	50	25	30	25	27	27	27	46	25	46

For the following analysis of the influence of phase 2 and 3 of the fire and the parameter variation to check the stability of the model only the distance of 80 miles is used because we assume that the main part of the beetles originate from the foothills of the Sierra Nevada. For phase 2 and phase 3 radiant heat fluxes of 7.1⋅10^−4^ to 7.2⋅10^−5^ W/m^2^ and 2.6⋅10^−4^ to 6.2⋅10^−5^ W/m^2^ were calculated at the foothills of the Sierra Nevada using the parameters in Table 4.

In order to get an impression of the stability of results due to changes in the input parameters, two case studies are defined: case “low radiation” with lower emissive power and higher losses and case “high radiation” with higher emissive power and lower losses, see Table 4. The pool diameter in phase 1 and 2 is set to the lowest and highest diameter for a 35.000 barrel and a 55.000 barrel tank. In phase 3 the diameter of the pool in the case “high radiation” is extrapolated to 75 m. The weather data used in “low radiation” were set to values that are valid for the morning hours resulting in a higher damping of the radiation. The reverse case is assumed for the case “high radiation”; here the values for the evening hours resulted in a lower damping of the radiation.

**Figure 17:**
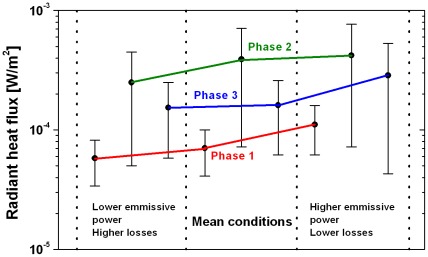
Comparison of the radiant heat flux 80 miles from Coalinga at the foothills of the Sierra Nevada for the cases “low radiation” with a lower emissive power and higher losses, “high radiation” with a higher emissive power and lower losses and the mean conditions, see **Fig. 16**.

The results of the case studies, shown in Fig. 17, are compared with the results of the mean conditions in Fig. 16. Compared with the radiant heat flux calculated in the case “mean conditions” the following deviations from the mean value of the “mean conditions” in the different phases became evident:
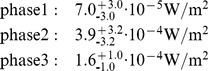
The result shows that deviations caused by variation of the input parameters are approximately the same or smaller than deviations generated by the respective fire models. As expected the radiant heat flux in phase 1 shows the lowest value whereas the transient phase 2 yields the highest radiant heat flux.

### The approach to the fire: calculation of flight speed and time

**Figure 18:**
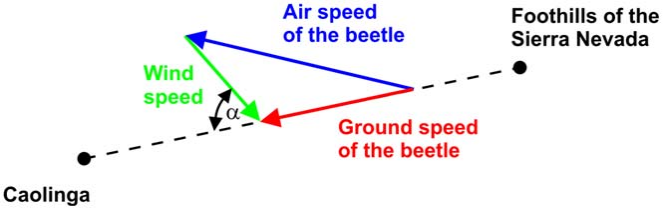
The ground speed vector of the beetle is the sum of the air speed vector and the wind speed vector.

Without human intervention a large wildfire burns for many days or even weeks. However the Coalinga oil-tank fire only lasted for three days. Therefore, the flight speed of *Melanophila* limits the distance from which a beetle can start without losing the IR signal on its way due to the extinguishing of the fire. The ground speed of the beetle is the vector sum of the wind speed and the species specific air speed of the beetle, see Fig. 18. As already mentioned in section “Input data for the calculations” the mean wind speed in August in the San Joaquin Valley (Fresno station) is 11.2 m/s ranging from 9.1 to 14.6 m/s over the year. The wind direction is mainly from the northwest to the southeast; this means that the angle α between the flight direction down from the foothills of the Sierra Nevada to Coalinga and the wind direction is about 56°. This strong cross wind had to be compensated by the beetle. Therefore the resulting ground speed is much lower than the maximal possible speed of flight which resulted in prolonged flight times.

However, the speed of flight of *Melanophila* beetles is unknown. Therefore we initially calculated a minimum flight speed necessary to reach the fire in time and compared it with speeds of flight known in insects [55].

For the calculation of the flying time it must be additionally taken into account that *Melanophila* beetles are diurnal and need ambient temperatures of about 25°C to initiate flight (K.-H. Apel, pers. com.). In August the sunrise in California is at 6:15 a.m. and the sunset is at 8:00 p.m.. Based on a typical daily temperature course in August where the beetles most probably would have swarmed was set from 8:00 a.m. to 8:00 p.m..

**Figure 19:**
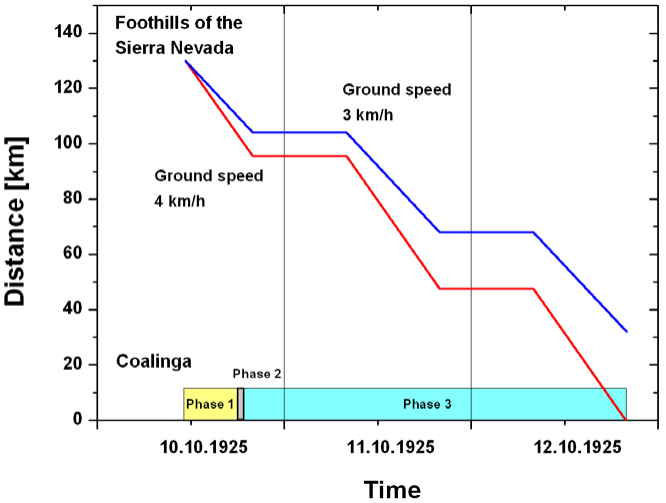
Time – distance diagram for the flight of the beetles from foothills of the Sierra Nevada to Coalinga. The outbreak of the fire and the three phases are indicated at the time axis. It is assumed that the beetles fly from 8:00 a.m. to 8:00 p.m. due to the swarming behaviour only by daylight and temperatures higher than approximately 20–25°C. For a ground speed of 4 km/h the beetle will reach the fire just in time before the fire burns out.

Assuming that the beetles had started immediately after the outbreak of the fire the time-distance diagram in Fig. 19 can be used to determine the minimum ground speed to about 4 km/h according to the conditions indicated.

The speed of flight of the beetle can be calculated according to the vector triangle in Fig. 18 with
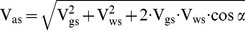
(24)with: V_as_ [km/h] : speed of flight of the beetle, V_gs_ [km/h] : ground speed of the beetle, V_ws_ [km/h] : wind speed, α : angle between V_as_ and V_ws_ according to Fig. 18

For a wind speed of 11.2 m/s, an angle α of 56° and ground speed of 1.1 m/s (4 km/h) Equation (24) yields an air speed of the beetle of 11.9 m/s (42.7 km/h). The variation of the wind speed of ±10% and the angle α±10% due to the uncertainties of the cross wind results in a range of the speed of flights from 13–11 m/s (46.9–38.1 km/h). This result indicates that the beetle must be a very fast and effective flyer. However, flight speeds in this range are not uncommon in insects and have been reported for members of different insect orders. The black cutworm *Agrotis ipsilon* (Noctuidae) is capable of flying with a speed up to 31 m/s (about 100 km/h, [56]); the horsefly *Hybomitra hinei* (Tabanidae) can fly even with a speed of 40 m/s (145 km/h, [57]). Both insects have body lengths of about 4 to 5 cm and, therefore, are much larger than *Melanophila* beetles. However, the small brown planthopper *Nilaparvata lugens* (Delphacidae) having only a body length of less than 5 mm, is able to fly with a speed of 22.4 m/s (80 km/h [58]). Therefore we conclude that it may possible that *Melanophila* beetles are also able to fly with a speed of 40 km/h or even higher.

If the beetle had started on the second day, 11.Oct.1925, i.e. in phase 3 of the fire, a ground speed of at least of 1.5 m/s (5.5 km/h) would be necessary to reach the fire in time. This yields an air speed of 12.1 m/s (43.5 km/h). Because this result is within the range due to wind speed variations mentioned above, it is not definitively necessary that the beetle started directly after the outbreak of the fire in phase 1. Without measurements of the possible air speeds of the beetle and more precise wind speeds it is not possible to decide on the triggering phase of the fire. Therefore all calculated radiant flux in the phases 1–3 could have been the triggering event.

### Thermal noise limits

The sensitivity of a sensor is limited by the sensor's noise level. In an uncooled thermal IR-sensor the prevailing ambient temperature inevitably causes thermal noise which defines the minimum noise level.

A schematic thermal circuit consists of a sensor target which is connected by a thermal link to a heat sink with a constant temperature T. In an uncooled IR-sensor the heat sink is at ambient temperature. The target can exchange power with the heat sink by radiation, thermal conduction and convective flow. When IR radiation is absorbed by the target an increase in temperature ΔT is induced. The minimum noise power results when only a radiative heat transfer between the target and the heat sink can take place [59], [60]. In case of pure black-body radiation with an emissivity of 1 and with ΔT≪T, the resulting noise power is:
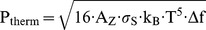
(25)With: A_Z_: cross-section of the target (active part of the sensillum), σ_S_ = 5.67040⋅10^−8^ W/m^2^ K^4^ : Stefan-Boltzmann constant, k_B_ = 1.3806504⋅10^−23^ J/K : Boltzmann constant, T: temperature of target and heat sink (T+ΔT≈T), Δf: bandwidth.

The minimum detectable radiant heat flux of an uncooled IR-sensor must be larger than the thermal noise (noise floor):
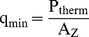
(26)Assuming a low-pass characteristics of the sensor, the bandwidth Δf can be estimated using the time constant τ of temperature change or alternatively the response time t_R_ of the sensillum [59].
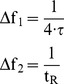
(27)For a cylindrical water-filled cavity with a very high-conductive wall material a simple estimate for the time constant was derived [61]

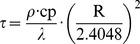
(28)With: ρ, cp, λ: density, heat capacity and heat conductivity of the fluid, R: diameter of the cavity

With a radius of the sensillum of R = 5 µm and the thermal properties of water as fluid a time constant τ = 29 µs, a bandwidth of Δf_1_ = 8,600 Hz and q_min_ = 5.8⋅10^−2^ W/m^2^ resulted. For a finite element model of the sensillum a more realistic time constant of τ = 640 µs was derived (Norbert Heß, DIAS Infrared Systems, Dresden, pers. communication). This yields a Δf_1_ = 390 Hz and q_min_ = 1.2⋅10^−2^ W/m^2^. The response time t_R_ of a sensillum was measured to 3–4 ms [18]. Accordingly, a bandwidth of Δf_2_ = 330–250 Hz and a q_min_ = 1.1⋅10^−2^–9.8⋅10^−3^ W/m^2^ resulted. The results show that the noise limit is about two orders of magnitude higher than the radiant heat fluxes probably perceived by the beetles at distances greater than 50 miles.

A solution with a narrower bandwidth will result when the beetle uses the frequencies of the flame pulsations (Helmut Budzier, Technische Universität Dresden, pers. communication). The use of a limited bandwidth focused on the pulsation frequencies seems even more probable because this allows the beetle to distinguish between a fire with a certain pulsation frequency and equally strong but steady radiant sources, e.g. radiating hot areas on the ground. Actually it was observed that the beetles approach by mistake hot spots like industrial furnaces. However, it is not experimentally investigated how the beetles distinguish between different heat sources.

Pulsation frequencies f_P_ even for large pool diameters were investigated by several authors. The pulsation frequency depends for a wide range of liquid fuels on the pool diameter:
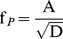
(29)With: f_P_ [Hz]: pulsation frequency, D [m]: pool diameter, A: constant factor

The factor A was determined to A = 1.5 for D<20 m [34], A = 1.6 for 2 m<D<50 m [62], A = 1.76 for 0.03 m<D<60 m [63], [64]. For pool diameters between 30 m–50 m as used herein pulsation frequencies between 0.2–0.3 Hz resulted. These frequencies are in good agreement with measurements of kerosene fires with a pool diameter of 30 mm and 50 m [65]. Here the measured frequency spectra showed the highest amplitudes below 1 Hz and pulsation frequencies with smaller amplitudes up to 2.5 Hz. Statements on the temporal stability of the pulsation frequencies could not be found in the present literature. Pulsation frequencies should also appear in forest fires. In [66] it was estimated that for a large fire with a diameter of 20 km a pulsation will occur every 20 minutes, that means at very low frequencies according to the large diameter, see Equation (2). Actually measured pulsation frequencies of forest fires are not known to the authors.

Based on this results the bandwidth Δf_3_ in case of observed pulsation frequencies is set to Δf_3_ = 10 Hz. This results in a minimum detectable radiant heat flux of about q_min_ = 2⋅10^−3^ W/m^2^. However, even with the smaller bandwidth the noise limit is one magnitude higher than the radiant heat fluxes probably detected by the beetles at large distances.

In general it is possible to detect signals which are hidden in noise, e.g. by adaptive signal processing [67], blind source separation [68] or phase space projection [69]. As will be discussed in section “Sensitivity of the IR receptors of *Melanophila* beetles” of the Discussion, the IR sensory system of *Melanophila* beetles could make use of stochastic resonance to detect the heat flux of the fire below the thermal noise level.

### Biological limits

In addition to the thermal noise limit a second limit exists: the minimal energy necessary to induce a suprathreshold response (i.e. at least a single action potential) in a single IR receptor. As mentioned, the real threshold of the *Melanophila* IR receptors is unknown. Therefore we use data well known for insect mechanoreceptors. The generation of an action potential in a highly sensitive insect hair mechanoreceptor requires a deformation of the dendritic tip of about 0.1 nm of the sensory cell innervating a wind sensitive hair cell in the cricket *Acheta domestica*. The energy required for this deformation is about 10^−19^ J [70]. In [22] even a minimum energy of 10^−20^ to 10^−21^ J is estimated for hair lengths between 100–1000 µm. Stochastic sampling, that means to sample a signal randomly instead using regular sampling intervals, most probably is used to achieve these ultra low sensitivities.

The radiant heat flux necessary for the energy increase in the sensillum is:
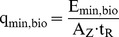
(30)With: E_min,bio_: minimal energy to produce an action potential, A_Z_: cross-section of the target (active part of the sensillum), t_R_: response time of the sensillum

Assuming a cross-section of the sensillum of A_Z_ = 8 ⋅ 10^−11^ m^2^ (10 µm diameter) and a response time of 3–4 ms until the first action potential is produced, Equation (30) yields a minimal radiant heat flux of 3 ⋅ 10^−7^–4 ⋅ 10^−7^ W/m^2^ for E_min,bio_ = 10^−19^ J and 3 ⋅ 10^−9^–4 ⋅ 10^−9^ W/m^2^ for E_min,bio_ = 10^−21^ J.

**Figure 20:**
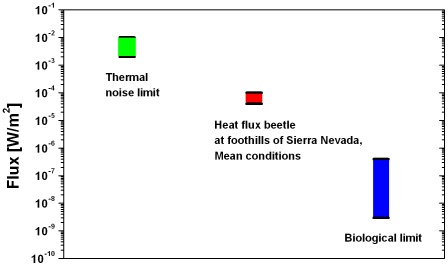
Comparison of the calculated heat flux perceived by the beetle at the foothills of the Sierra Nevada (mean conditions) with the thermal noise limit, depending on assumed bandwidth, and biological limit, depending on the minimal energy resolution of the sensillum.

Fig. 20 compares the estimated thermal noise limit and the biological limit with the calculated radiant heat flux the beetle probably detected at the foothills of the Sierra Nevada (mean conditions, phase 1). If one accepts that the beetle can detect radiant heat fluxes below the thermal noise limit due to the use of stochastic resonance, see section “Sensitivity of the IR receptors of *Melanophila* beetles”, then there is a sufficient safety reserve regarding the biological limit, even with the lower energy resolution of 10^−19^ J.

### Sensitivity thresholds based on the modeling of the Coalinga oil-tank fire

As explained in detail in the Discussion (cf. section “Sources of the beetles”) it can be assumed that the majority of the beetles observed in Coalinga originated from the forests which cover the western mountain foothills of the Sierra Nevada. Additionally, there is evidence that a smaller fraction of beetles approached the fire from the wooded region of San Benito Mountain Natural Area.

Table 5 gives the distances from Coalinga to these two localities and a compilation of the calculated heat fluxes and energy levels at single IR receptors. Based on the estimation of the necessary flight time calculated in section “The approach to the fire: Calculation of flight speed and time” it can be concluded that beetles which detected the fire from a distance of 130 km and finally reached Coalinga had perceived the low radiation intensity during the initial phase 1 of the fire. Consequently it has to be postulated that the sensitivity threshold of the IR receptors of *Melanophila* beetles is in the range of 1.3×10^−4^ to 4.1×10^−5^ W/m^2^ which corresponds to energy levels at a single IR sensillum between 1.3×10^−17^ J. Depending on the phase of the fire, heat fluxes and energy levels at single receptors are correspondingly higher at San Benito Mountain.

**Table 5:** Possible detection ranges of the oil-tank fire by biological and technical IR sensors.

Distance km	Locality/data basis	Heat flux [W/m^2^] = sensitivity threshold	Lowest energy level at single receptor [J]	Source
2.0–2.5	Data published by Evans	0.6	2×10^−13^	Evans 1964, 1966
25	San Benito Mountain AreaPhase 1/3	5×10^−3^ to 2.1×10^−3^3×10^−2^ to 7.6×10^−3^	7×10^−16^	This paper
130	Western foothills of Sierra NevadaPhase 1	1.3×10^−4^ to 4.1×10^−5^	1.3×10^−17^	This paper
up to 35	Maximal detection distance with pyroelectric IR sensor	2.4×10^−3^	n. a.	Perkin-Elmer Sensor's Brochure 2011

Additionally, we have calculated the maximal distance from which a beetle could have detected the tank-fire if the threshold would be 0.6 W/m^2^ as published by Evans already in the sixties of the last century. Finally, we included a threshold of a current high sensitivity uncooled IR sensor and calculated the maximal distance from which detection of the Coalinga tank fire would have been possible with such a sensor (cf. Table 5).

## Discussion

### Source of the beetles

As already proposed by Palm [71], Apel has provided strong experimental evidences that wood boring *Melanophila* larvae essentially depend on freshly burnt wood [1], [72]. Apel also showed that the highly pyrophilous species *Melanophila acuminata* perform extensive mass-breeding on burnt areas. The author extrapolated that the burnt logs on a 1.8 ha (18.000 m^2^) pine plantation in eastern Germany must have contained about 300,000 larvae of *Melanophila acuminata*. The plantation was situated within a larger burnt area of 20 ha. Thus at least 10,000 females of *Melanophila acuminata* had been attracted by the fire and deposited their eggs into the trees on the 1.8 ha plot [72]. The challenging task for every newly hatched *Melanophila* beetle, therefore, is to find a fire. Up to now nothing is known about the dispersal behaviour of *Melanophila* beetles. Mark and recapture experiments have not been done so far. The majority of adult buprestid beetles is diurnal, sun-loving, and oligophagous [14]. This is also true for *Melanophila* beetles (corroborated by many own observations). Thus dispersal will be influenced by the daily activity and flight behaviour as shown for the buprestid beetle *Capnodis tenebrionis*, which spreads from one orchard to another [73]. However, the situation regarding the finding of trees freshly killed by a fire is something special because the outbreak of a fire is unpredictable. We propose that as long as the beetles do not receive any sensory stimulus from a fire they will undertake extensive search flights thereby disappearing from the old burnt area. However, most probably these flights will not be totally random. If the burnt area is situated within a larger forest, beetles most probably will disperse within some days or weeks all over the forest where a new fire may start. However, the smaller the forest is, the larger the need will be to leave the forest. Finally beetles will depart from the outskirts of the original forest. We propose that beetles try to overcome unwooded regions to reach another wooded area. Thus a high population density of beetles on unwooded terrain is rather unlikely. As already proposed by Van Dyke [74], the majority of the beetles which approached the tank-fire must have originated from nearby forests.

**Figure 21:**
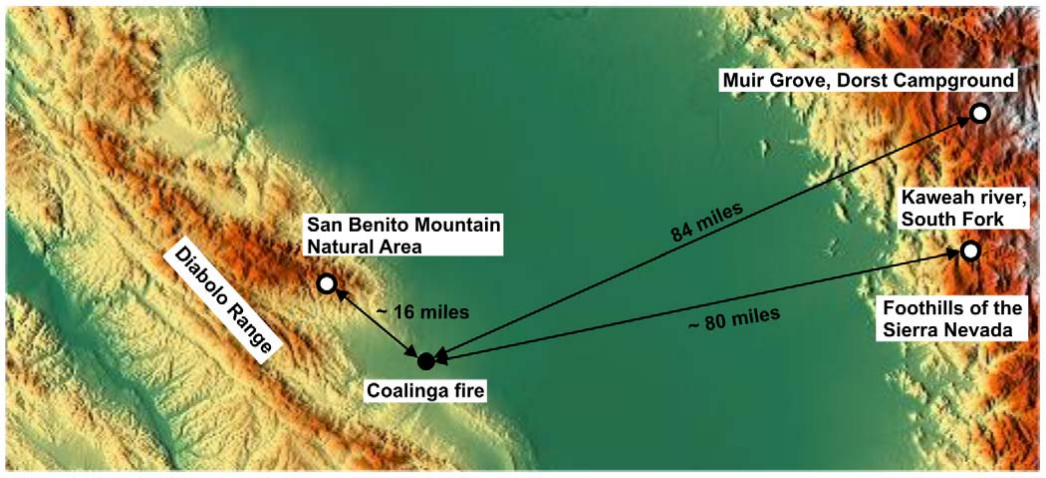
Identification of the distances between the Coalinga fire (1925) and the fire at Muir Grove (1923), the fire at the South Fork of Kaweah River (1924) with the foothills of the Sierra Nevada and the San Benito Mountain Natural Area. Map based on [98].

However, Coalinga is situated in the Central Valley of California (San Joaquin Valley) where conditions are too dry and arid for trees. In general, today's conditions are still comparable to the situation in 1925 because since 1925 and today no significant forest disturbances between Coalinga and the present forested areas took place (Tom Coleman, US Forest Service, pers. communication). The next forested area is the San Benito Mountain Natural Area as part of the Diabolo Range. The distance between the south eastern edge of the woods around San Benito Mountain and Coalinga is about 25 km (Fig. 21). However, for several reasons it is rather unlikely that all beetles which were observed at the Coalinga fire stemmed from the San Benito Mountain Area. Even with the generous help of Ryan E. O'Dell and Erik C. Zaborsky from the Bureau of Land Management (Hollister Field Office, CA) who provided us with a GIS (Geographic Information System) data layer including a “Fire History of California” layer starting in 1878, we were not able to identify a forest fire in the San Benito Mountain Area one or two years before the Coalinga tank-fire. Additionally, Ray Iddings of Three Rocks Research in Fresno, CA, provided us with detailed information about historic fires in the San Benito Mountain Area in the beginning of the 20^th^ century. Again, no indications for fires in 1923 or 1924 were found. A possible reason that reports about larger fires in the San Benito Mountain Area are rare may be that the terrain is very rough consisting of high ridges and steep-sided canyons. Therefore, the spreading of a fire is prevented by many natural fire breaks and the emergence of larger fires is hindered. Accordingly, the San Benito Mountain area as a potential source for the beetles was not mentioned in the report of Van Dyke. However, the summer of 1924 (one year before the oil-tank blaze) was characterized by one of the worst fire seasons in California: after a two year drought about 1 million acres of forest burned [75]. Therefore we speculate that a given percentage of the beetles may have originated from a few smaller burnt plots in the San Benito Mountain Area. However, the majority of beetles most probably stemmed from other forests.

In the report of Van Dyke two distances are mentioned from which the beetles most probably had approached the fire. The smaller value is 50 miles (80 km) which corresponds to the distance between Coalinga and the outskirts of the western forests covering the coastal Santa Lucia Range. The forests extend from Monterey in the north down to San Luis Obispo in the south. Although we were not able to identify forest fires in 1924 or 1925 we are convinced that some fires had occurred in the coastal forests in these years. However, for all beetles which sojourned in the coastal forests the Coalinga fire was completely shadowed by the towering ridges of the unwooded Diabolo Range separating Coalinga from the Santa Lucia Range. So only beetles which already had overcome the 600–800 m high ridges of the Diabolo Range, had a chance to become aware of the tank fire by IR radiation (Fig. 21). Thus we propose that due to the geographical situation only a minority of beetles may have originated from the coastal western forests.

The second distance between Coalinga and a larger forest mentioned in the report of Van Dyke is 100 miles. This corresponds to the distance between Coalinga and the forested western foothills of the Sierra Nevada: namely the western edges of the Sequoia and Kings Canyon National Park.

From our perspective, these eastern forests are the most promising sources of beetles found at the Coalinga tank fire. So we were able to identify a 40 ha fire in 1924 in the Sequoia and Kings Canyon National Park in oak woodland, chaparral, and evergreen hardwood forest in the south fork of the Kaweah River about 130 km east of Coalinga (information provided by Tony Caprio, Sequoia and Kings Canyon National Parks). Additionally, a 40 ha fire from 1923 which occurred in the Sequoia National Park in mixed conifer forest in the area of the Muir Grove of giant sequoias near Dorst campground (Fig. 21) could have been a source of beetles because some percentage of the larvae have a two-year developmental cycle (fire information also provided by Tony Caprio). It can be assumed that after a pronounced mass breeding which was initiated by the summer fires in 1924, millions of beetles hatched one year later and distributed all over the extensive forests of the Sierra Nevada. Especially beetles which stayed in the woods on the western foothills of the Sierra Nevada had a good chance to perceive IR radiation form the tank fire: the flat San Joaquin plain permits an unhindered view up to Coalinga. We propose that a considerable amount of beetles stemmed from these areas.

### Infrared radiation as cue for remote fire detection

The next step during a search flight will be the perception of fire indicating stimuli (cf. Table 1). Initially it was speculated that the beetles were guided by olfactory cues to a fire and that the thoracic pit organs are chemoreceptors [10], [15]. However, in later behavioural experiments in an olfactometer *Melanophila acuminata* could not be attracted by smoke [17]. In our own experiments it was also not possible to arouse resting *Melanophila acuminata* by presenting freshly produced smoke at different concentrations from deciduous and coniferous trees. We tested low as well as high concentrations and set ambient temperatures to about 25°C which guaranteed that beetles were fully agile and could take off at any time. Additionally, the reproductive status of the beetles was taken into consideration (beetles used were unmated and at least one week old).

These results lead to the general question whether the smell of smoke is a suitable cue for the detection of forest fires. Because of two reasons we speculate that this may be dubious. First we analysed many aerial pictures of smoke plumes of forest fires which are easy to retrieve by the internet. As a general result nearly all smoke plumes can be divided into two zones. In the first zone next to the source of the fire the smoke is driven away for many kilometres by the present wind within a relatively narrow angle. Consequently, all beetles which stay in the much larger external angle of a smoke plume have no chance to become aware of the fire by olfaction even if they are nearby. This is especially disadvantageous because this first zone of the plume is mostly still situated over forested areas where most beetles will sojourn. The second reason is founded by the characteristics of the second zone of the smoke plume. This zone which for the case of large fires can be several days old is much more extended and covers a much larger territory. Often a gradient in smoke concentration with a constant increase towards the source is no longer present. Additionally, wind direction is extensively influenced by the topography of the landscape and, therefore, may be different at a distance of 100 or 200 km away from the fire. A beetle performing upwind flight behaviour may reach the border of the smoke plume somewhere and – without a concentration gradient – will have no chance to decide in which direction the flight has to be continued. As a result, we postulate that odour guided upwind flight behaviour is not a promising strategy to find a forest fire from larger distances.

Theoretically, there might be the possibility that beetles can see a smoke plume from some distance. However, clouds may mislead the beetles which most probably cannot waste energy to fly a couple of kilometres just on the strength of a hunch. The glow of the fire can be only seen at night. As already mentioned, *Melanophila* beetles are diurnal and therefore it is very unlikely that they detect a fire from larger distanced by seeing flames.

Looking at Table 1 the only cue remaining is IR radiation. A big advantage of IR radiation is that once given off by the fire it propagates - unaffected by weather conditions except the damping influence of increasing humidity - through the atmosphere within two atmospheric windows. A clear gradient also exists because intensity monotonously decreases with the square of the distance from a source. If the sensitivity of an IR receptor is high enough, it is no problem to detect a fire even from the outer space.

### Sensitivity of the IR receptors of *Melanophila* beetles

Basically the sensitivity of the IR receptors in the thoracic pit organs of *Melanophila* beetles is unknown. Consequently, a lot of highly contradictory information with regard to possible detection distances for fires can be found in the literature. In brief, published detection ranges vary between 160 km [12] and, as claimed recently, about 50 m [76].

Relatively few attempts have been made to determine the sensitivity threshold experimentally. 45 years ago, Evans performed first behavioural experiments and published a threshold of 0.6 W/m^2^ [16], [17]. However, these data have to be considered critically. In the experiments beetles did not fly but were hanging down from a piece of aluminium foil to which they were glued with the pronotum. The monitored behavioural response to IR radiation was a “twitching” (i.e. a sideway back- and forward movement) of the antenna ipsilateral to the irradiated pit organ. In an attempt to reproduce these experiments we failed to elicit the described antennal twitching by diffuse broadband IR radiation of different intensities. It was easy, however, to trigger ipsilateral antennal twitching by strong flashes of visible light. It is possible that the described antennal movement represents an unspecific startle response to protect the antenna by briefly hiding it under the head. Because in Evan's experiments IR radiation was focused onto the pit organs it seems possible that IR radiation with intensities down to about 0.6 W/m^2^ caused overstimulation of the IR receptors to which the beetles responded with antennal twitching. Below 0.6 W/m^2^ behavioural responses to IR radiation may be detectable only in flying beetles as slight deviations from a straightforward flight path. Thus monitoring an unspecific twitching of the antenna seems not suitable to determine the sensitivity threshold of the IR receptors. Furthermore the importance of a unilateral antennal twitching in flight remains dubious. We doubt that the described antennal twitching occurs during flight at all because such a sudden symmetric movement most probably will negatively affect flight stability. Based on his results Evans has calculated that a beetle should be able to detect a hypothetical 20 hectare (200.000 m^2^) fire from a distance of up to 5 kilometres [16]. The more recent work in [76] states that *Melanophila* beetles cannot make use of their IR organs to detect a fire; although no new data seem to support this claim.

More than 30 years after Evans experiments first electrophysiological experiments were made. All recordings were made extracellularly either by metal electrodes or by small hook electrodes placed around the connectives between the pro- and mesothoracic ganglia [18], [19], [77], [78]. The lowest threshold based on electrophysiological recordings published so far is 5 W/m^2^ and is based on recordings with metal electrodes inserted in the cuticle at the bottom of the pit organ [18]. However, in these experiments the metal electrode was placed directly next to the sensillum recorded from. Most probably the thermal properties of the cuticle around the insertion site including the minute sphere of the IR sensillum were significantly altered because considerable amounts of heat were withdrawn from the cuticle. A serious reduction in sensitivity can be expected. The authors presented a rough calculation suggesting that beetles should be able to detect a hypothetical 10 hectare fire from a distance of 12 kilometres.

In summary, the lowest sensitivity threshold ever reported for the *Melanophila* IR sensilla still is the doubtful 0.6 W/m^2^ published in the sixties of the last century which has the uncertainties discussed above. This threshold would have permitted a detection of the oil-tank fire from a distance of only 10 km (cf. Table 5). But within a 10 km radius around the tank the area was unforested and - as already discussed - could not have been the source of the enormous number of beetles observed at the fire.

The results of our simulation show that the heat fluxes at the southern outskirts of the forest around San Benito Mountain and especially at the western slopes of the Sierra Nevada are several orders of magnitude lower. Accordingly the resulting energy levels at a single IR sensillum are up to 4 orders of magnitude lower (Table 5). Our results suggest that the detection of the IR from the tank fire could have been possible, when implemented with suitable, putative neuronal amplifying mechanisms that have been described in the literature.

In this context it is important to realize that the IR sensilla of *Melanophila* beetles are innervated by ciliary mechanoreceptors. In general, specialized arthropod mechanoreceptors like flow sensors show the highest sensitivities known in biological sensors. This has been studied in great detail in spider trichobothria [79], [80] and in filiform hairs in insects [22], [23]. In filiform hairs in crickets which are specialized for sensing air flow the minimum amount of mechanical energy for the generation of an action potential is in the order of k_B_T (k_B_: Boltzmann constant, T: temperature, 4×10^−21^ J at 300°C; [22]. At threshold, the trichobothrium works near the thermal noise of Brownian motion and, therefore, operates at the limit of the physically possible [23], [80].

Additionally, signals can be detected which are three orders of magnitude lower than the broadband ciliary displacement noise [81]. This is of special interest because at least the heat fluxes which reached the edges of the forests of the slopes of the Sierra Nevada are deeply buried in thermal noise (cf. Fig. 20). In principle the array of about 70–90 receptors situated in each IR organ can increase the sensitivity by summation of the responses of many receptors. This has been shown e.g. for olfactory receptors which can be found in great numbers of several thousand sensilla on the insect antenna. Multiple sensors allow the detection of much lower signal amplitudes compared to a single sensor because central neurons can sum the responses from many peripheral receptors thus increasing the signal-to-noise ratio [82]. Under certain condition signals which are far below the thermal noise level can still be detected by so-called stochastic resonance. Stochastic resonance allows the enhancement of weak periodic signals by a certain “resonant” noise intensity [83]. It can be used in technical systems [84], but has also been described in biological systems [85]. For the application of stochastic resonance three requirements must be met: a weak periodic signal below treshold, a noise level that is larger than the periodic signal (except the signal is close to threshold) and the system has to be nonlinear, e. g. due to a level or threshold the signal has to pass before it will become detectable by the sensor. All these requirements are met very well for sensory cells like mechanoreceptors where a preset amplitude of the receptor potential has to be reached before an action potential is generated. Accordingly, several investigations have shown that for hair cells in the inner ear the addition of noise leads to an improvement in the output signal to noise ratio [86], [87], [88]. Experiments with hydrodynamically sensitive mechanoreceptors hair cells located in the tailfans of crayfish *Procambarus clarkii* showed that the detection of weak signals can be enhanced by an optimal level of external noise in single sensory neurons [89]. Experiments on the cercal system of the cricket *Acheta domestica* demonstrated that a significant degree of encoding enhancement can be achieved by stochastic resonance [90]. However, also in the peripheral electroreceptors of the paddlefish *Polyodon spathula* an increased sensitivity due to stochastic resonance was observed [91] and electrical or mechanical noise enhances the ability of humans to detect subthreshold mechanical cutaneous stimuli [92].

Although experimental proof demonstrating the IR sensillum of *Melanophila* beetles uses stochastic resonance is still missing, all the reports described in the *Introduction* suggest that the beetles are able to detect radiant heat fluxes below the thermal noise limit which points to the use of stochastic resonance. Currently no sensory system is known which is capable of detecting signals 2–3 times below the thermal limit; nevertheless the use of stochastic resonance seems reasonable.

Another mechanism to increase the sensitivity in a mechanosensory system like hearing organs is based on the active contribution of motile mechanosensitive cells which feed mechanical energy into the oscillations inside an ear. In vertebrate ears, hair cells are capable of inducing vibrations of the basilar membrane by intrinsic molecular motors [93], [94], [95]. In the antennal hearing organs of mosquitos and the fruit fly *Drosophila* the mechanosensory cells of the chordotonal organs generate self-oscillations of the distal parts of the antenna which serve as sound receivers [96], [97]. However, due to the apparent lack of moveable components in an IR sensillum it is disputable whether the mechanosensory cell is capable of enhancing the sensitivity by an intrinsic motility.

### Conclusions and suggestions for further work

In our study we have compiled several arguments suggesting that beetles of the genus *Melanophila* use their IR receptors for the detection of distant fires. Based on these considerations we proposed that beetles detected the Coalinga oil tank fire by IR reception and determined possible thresholds for the sensitivity of the IR receptors. These thresholds, however, have to be corroborated by additional behavioural and electrophysiological experiments combined with calibrated IR lab sources. If it should turn out that the sensitivity of the *Melanophila* IR receptors really is within the range revealed by our simulation, photomechanic IR sensilla must have a greater sensitivity than current uncooled IR sensors used for the detection of mid-IR radiation. This is of special interest because considerable efforts are undertaken to close the gap in sensitivity between highly sensitive semiconductor based IR sensors (e.g. MCT quantum sensors) which have to be cooled and less sensitive uncooled thermal IR sensors like pyroelectric IR sensors and microbolometers. According to our calculations the sensitivity of the *Melanophila* IR sensilla should be in between the sensitivities of these two groups. In ultra-sensitive filiform hairs of insects, the long bristle is crucial to convert energy form the flow field to the sensory cell. Thus it has to be postulated that the unique cuticular spheres which can be found instead of a bristle in a *Melanophila* IR sensillum are also capable of a highly efficient conversion of the energy of absorbed IR photons into micromechanical action instantaneously perceived by the mechanosensitive dendrite.

Therefore, further investigation of the mechanisms of thermo/mechanical energy conversion managed by the cuticular apparatus of the *Melanophila* IR sensilla seems to be highly rewarding with view to the development of new sensitive photomechanic IR sensors.
